# Aspirin use and lung cancer in men

**DOI:** 10.1038/sj.bjc.6601343

**Published:** 2003-10-28

**Authors:** C N Holick, D S Michaud, M F Leitzmann, W C Willett, E Giovannucci

**Affiliations:** 1 Department of Nutrition, Harvard School of Public Health, 677 Huntington Avenue, Boston, MA 02115, USA; 2 Department of Epidemiology, Harvard School of Public Health, 677 Huntington Avenue, Boston, MA 02115, USA; 3 Nutritional Epidemiology Branch, Division of Cancer Epidemiology and Genetics, National Cancer Institute, Bethesda, MD 20892, USA; 4 Channing Laboratory, Department of Medicine, Brigham and Women's Hospital, Boston, MA 02115, USA; 5 Harvard Medical School, Boston, MA 02115, USA

**Keywords:** aspirin, lung cancer, chemoprevention, prospective studies, epidemiology

## Abstract

We examined prospectively the relation between regular aspirin use and lung cancer risk in the Health Professionals Follow-Up Study. Of 49 383 US men aged 40–75 years who completed biennial self-administered questionnaires that assessed aspirin use beginning in 1986, 328 developed lung cancer during 601 453 person-years of follow-up through 31 December 2000. No information on aspirin dose was available. Controlling for current age, smoking status, and age at starting to smoke regularly, the relative risk (RR) of total lung cancer for regular users of aspirin (twice or more per week) at baseline compared to nonusers was 1.13 (95% confidence interval (CI) =0.89–1.43). Results were similar for non-small-cell lung cancer (RR=1.16; 95% CI=0.88–1.54). No apparent dose-dependent association was observed for the frequency of aspirin use and lung cancer risk (*P* for trend=0.64), and results remained null when consistent use of aspirin over time was examined. These findings do not suggest that regular aspirin use is associated with a reduced lung cancer risk.

Evidence from animal studies suggests that aspirin and possibly other nonsteroidal anti-inflammatory drugs (NSAIDs) including cyclooxygenase-2 (COX-2) inhibitors may influence the development and progression of lung cancer (Duperron and Castonguay, 1997; Rioux and Castonguay, 1998; Yao *et al*, 2000). As COX-2 enzymes are constitutively expressed in lung neoplastic tissue (Xu, 2002), reduced synthesis of prostaglandins from arachidonic acid by inhibition of COX-2 enzymes may have preventive or therapeutic effects in lung carcinogenesis.

Clinical and epidemiological studies, however, have not consistently supported for the hypothesis that regular use of aspirin and other NSAIDs reduces lung cancer incidence but are limited in several key areas. These include residual confounding due to broad categories of smoking habits (Paganini-Hill *et al*, 1989; Schreinemachers and Everson, 1994; Langman *et al*, 2000; Akhmedkhanov *et al*, 2002; Moysich *et al*, 2002); misclassification due to a single assessment of aspirin use (Paganini-Hill *et al*, 1989; Schreinemachers and Everson, 1994; Harris *et al*., 2002); and small number of lung cancer cases observed (Peto *et al*, 1988; Paganini-Hill *et al*, 1989; Schreinemachers and Everson, 1994; Akhmedkhanov *et al*, 2002).

Given the limitations of previous literature and uncertainty regarding the protection of aspirin against lung cancer risk, we examined the association between regular use of aspirin and lung cancer incidence in a large cohort of US. male health professionals with long-term aspirin use and detailed information on smoking history.

## MATERIALS AND METHODS

### Study population

The Health Professionals Follow-Up Study (HPFS) is an ongoing prospective cohort study of 51 529 US male dentists, optometrists, osteopaths, podiatrists, pharmacists, and veterinarians aged 40–75 years. The primary objective of the study is to evaluate a series of hypotheses relating nutritional and lifestyle factors to the incidence of cancer, heart disease, and other major diseases. At baseline in 1986, enrollees returned a mailed questionnaire that assessed information on lifestyle factors, aspirin and other NSAID use, diet, and medical and smoking histories. Follow-up questionnaires are mailed biennially to the entire cohort to update information on potential risk factors and ascertain new cases of disease.

### Assessment of aspirin use

Regular use of aspirin (e.g. Anacin, Bufferin, Alka-Seltzer), acetaminophen (e.g. Tylenol), and other NSAIDs (e.g. Motrin, Indocin, Naprosyn, Dolobid) separately defined as two or more times per week was ascertained in 1986 and updated every 2 years thereafter. More detailed questions on aspirin use began in the 1992 questionnaire. Frequency of aspirin use was assessed by the average number of days each month aspirin was taken (none, 1–4, 5–14, 15–21, or 22 or more). Information on aspirin dose was not available.

Reasons for aspirin use were assessed in 1993 by a supplementary questionnaire sent to a random sample of 211 participants, who reported taking aspirin from 1986 to 1990. Of the 211 men, 185 (88%) responded and reported one or more of the following reasons: cardiovascular disease, 25%; to decrease risk for cardiovascular disease, 58%; headache, 25%; joint or musculoskeletal pain, 33%; and other or unknown reasons, 7%. We were unable to examine long-term use of acetaminophen and other NSAIDs separately because of the small number of men who used them regularly (approximately 6% of the cohort).

### Identification of lung cancer cases

On each biennial questionnaire participants were asked whether they had been diagnosed with lung cancer during the previous 2 years. The follow-up rate with respect to the incidence of cancer was 96% of the total possible person-years. After receiving permission from identified cases (or next of kin for decedents), hospital records and pathology reports were obtained and reviewed by a physician for histological confirmation. Information on lung cancer cell type was available for approximately 80%. Deaths in the cohort were ascertained through family members and the National Death Index (Stampfer *et al*, 1984). Cases diagnosed between date of 1986 questionnaire return and 31 December 2000 were included in this report (*n*=328).

### Statistical analysis

Follow-up for each participant was calculated from the date of return of the 1986 questionnaire until the date of lung cancer diagnosis, date of death from any cause, or 31 December 2000, which ever came first. At baseline in 1986, we excluded 2069 men who reported a history of cancer other than nonmelanoma skin cancer by 1986, 40 men who incorrectly completed the questionnaire, and 37 men who later wished to be removed from the cohort. Only those with complete information on aspirin use at baseline were included in the analyses. The analytic cohort consisted of 49 383 men representing 601 453 person-years of follow-up. Cox proportional hazards models were used to estimate relative risks (RRs) and 95% confidence intervals (CI).

To take account for changes in aspirin use over time and best represent long-term use of aspirin, our analyses were conducted using consistent aspirin use. For example, lung cancer from 1986 through 2000 was related to aspirin use reported on the 1986 questionnaire, that from 1988 through 2000 to consistent use reported in the 1986 and 1988 questionnaires, that from 1990 through 2000 use reported on the 1986, 1988, and 1990 questionnaires, and that from 1992 through 2000 use reported on the 1986, 1988, 1990, and 1992 questionnaires. Consistent aspirin users were compared to nonusers (i.e., participants who consistently reported no aspirin use during the same specified time periods).

Potential confounders were specified *a priori* based on a review of putative risk factors for lung cancer and included age (year); marital status; body mass index (kg m^−2^); use of multivitamins, vitamin A, vitamin C, vitamin E, *β*-carotene, and selenium supplements (yes or no); intake of fruit and vegetables; energy-adjusted *α*-carotene, *β*-carotene, lycopene, lutein/zeaxanthin, and *β*-cryptoxanthin; alcohol use; and family history of lung cancer. Smoking history was categorised as the combination of age at starting to smoke regularly (less than 15, 15–19, 20–29, and more than 30 years old) and smoking status, which included current smoking (1–4, 5–14, 15–24, 25–34, 35–44, and 45 or more cigarettes per day), past smoking with time since quitting (less than 10 years and 10 or more years), and never smoked. Multivariate models included current age, age at starting to smoke regularly, and smoking status (included time since quitting and dose), plus the additional confounders mentioned previously that were assessed by evaluating whether their inclusion into the multivariate model changed the risk estimate by more than 10%. Additional analyses were restricted to non-small-cell lung cancer (NSCLC) (squamous carcinoma, adenocarcinoma, and large cell carcinoma). Potential interaction by smoking status category (never, past, and current separately) and baseline aspirin use was assessed using a cross-product term in multivariate models. Tests of linear trends for increasing categories of aspirin use were conducted by assigning the median value of aspirin use for categories and treating these as a single continuous variable. Reported *P*-values are based on two-sided tests.

## RESULTS

At baseline in 1986, about one-third of men reported regular aspirin use defined as two or more tablets per week (Table 1

**Table 1:** Characteristics^a^ (means and proportions) of the HPFS cohort by aspirin use at baseline in 1986

	**Aspirin**	
**Characteristic**	**Nonuse**	**Use^b^**
Participants (*n*)	34 841	14 542
Age (years)	53.7	56.4
Height (in)	70.0	70.1
Body mass index (kg m^−2^)	24.9	25.1

*Smoking history*^c^		
Smoking status (%)		
Never	45.8	41.9
Past (years)		
<10	11.4	14.9
10+	27.7	31.9
Current (cigarettes per day)		
1–4	0.9	1.1
5–14	1.5	1.9
15–24	3.2	3.1
25–34	1.4	1.6
35–44	1.0	1.2
45+	0.4	0.5
Age (years) at starting to smoke (%)		
<15	5.7	6.9
15–19	18.9	23.4
20–29	20.8	23.6
30+	47.6	44.0
Years since quitting	8.3	8.1

*Intakes (per day)*		
Fruit and vegetable	5.3	5.3
*β*-Carotene (*μ*g)	5150	5039
*α*-Carotene (*μ*g)	949	918
Lycopene (*μ*g)	10 374	10 321
Multivitamin use (%)	39.4	50.0
*Supplement use (per day)*		
Vitamin C (*μ*g)	57.6	67.7
Vitamin E (IU)	17.6	22.7
Vitamin A (IU)	7.9	9.3
Selenium (*μ*g)	6.2	7.5
*β*-Carotene^d^ (*μ*g)	1.9	2.6
		
Family history of lung cancer (%)	5.7	6.0
*Disease history* (%)		
Asthma	5.2	5.4
Emphysema or chronic bronchitis^e^	1.4	1.6
Rheumatoid arthritis	2.0	3.1
Other arthritis (e.g. degenerative)	7.3	12.2

a Standardised to the age distribution of the study population.

b Aspirin use was defined as aspirin use two or more times per week.

c Values do not add up to 100% because of missing values.

d Use of supplement on a regular basis.

e Data from the 1996 follow-up questionnaire.

). Aspirin users tended to be slightly older, to have smoked, and to have started smoking at an earlier age. Dietary patterns were very similar except for multivitamin and supplement use, reflecting the tendency of aspirin users to also take supplements. Family history of lung cancer was similar among users and nonusers of aspirin.

Overall, we observed no significant association between aspirin use at baseline and total lung cancer incidence (Table 2

**Table 2:** Relative risk of lung cancer by aspirin use in the HPFS, 1986–2000

	**Aspirin use^a^**			
**Variable**	**1986**	**1986+1988**	**1986+1988+1990**	**1986+1988+1990+1992**
Follow-up period^b^	1986–2000	1988–2000	1990–2000	1992–2000

*Total cases*				
Nonusers, *cases/person-years*	204/428 688	96/202 457	66/126 065	22/44 436
Users, *cases/person-years*	124/172 765	64/93 431	41/52 730	28/37 478
Age-adjusted RR^c^ (95% CI)	1.19 (0.95–1.49)	1.11 (0.80–1.53)	1.07 (0.72–1.60)	1.18 (0.66–2.10)
Multivariate RR^d^ (95% CI)	1.13 (0.89–1.43)	0.98 (0.70–1.36)	0.88 (0.58–1.34)	0.89 (0.47–1.67)

*Non-small-cell carcinomas*				
Nonusers, *cases/person-years*	140/428 717	69/202 466	45/126 073	13/44 441
Users, *cases/person-years*	87/172 789	49/93 441	33/52 733	24/37 479
Age-adjusted RR^c^ (95% CI)	1.25 (0.95–1.64)	1.20 (0.82–1.74)	1.27 (0.80–2.01)	1.76 (0.88–3.54)
Multivariate RR^d^ (95% CI)	1.16 (0.88–1.54)	1.02 (0.69–1.49)	0.98 (0.61–1.58)	1.10 (0.52–2.35)

a Aspirin use was defined as aspirin use two or more times per week. Users consistently reported regular aspirin use (1986; 1986 and 1988; 1986, 1988, and 1990; 1986, 1988, 1990, and 1992); nonusers consistently reported no aspirin use during the specified time periods.

b Separate proportional hazards models were analysed for each of the four follow-up periods.

c Proportional hazards models adjusted for current age.

d Proportional hazards model adjusted for current age, age at started to smoke regularly, and smoking status (includes dose and time since quitting).

). Age-adjusted associations were attenuated after adjustment for age at starting to smoke regularly and smoking status but did not change appreciably after additional inclusion of the other covariates. The association between duration of aspirin use and lung cancer risk became slightly inverse with evidence of more consistent aspirin use (consecutive RRs of 1.13, 0.98, 0.88, and 0.89), but the results were not statistically significant. After adjustment for current age, age at starting to smoke regularly, and smoking status the RR of total lung cancer for users of acetaminophen and other NSAIDs separately at baseline compared to nonusers was 1.26 (95% CI=0.81–1.96) and 1.07 (95% CI=0.69–1.66), respectively (*N*=23 cases among users for both compounds).

Previous studies (Akhmedkhanov *et al*, 2002; Moysich *et al*, 2002) have examined the association between aspirin use and NSCLC. We limited the analysis to NSCLC (227 cases) and compared baseline aspirin use with nonuse of aspirin (Table 2); the multivariate risk was 1.16 (95% CI=0.88–1.54). Furthermore, no evidence of an association was observed with increasing consistency of aspirin use and risk of NSCLC.

We also evaluated the association between frequency of aspirin use, first assessed in 1992, and lung cancer risk, but no dose-dependent association was observed. The multivariate RRs of lung cancer for increasing frequency of aspirin use (0–4, 5–21, and 22 or more days per month) were 1.00, 0.62, and 1.21 (95% CI=0.68–2.16), respectively (*P* for trend=0.64). Similar nonsignificant associations for any given amount of aspirin use were observed for the non-small-cell types (*P* for trend=0.40).

No evidence of interaction was observed between categories of smoking status and baseline aspirin use for lung cancer risk. After controlling for age, the RR of lung cancer for aspirin users *vs* nonusers among never smokers was 1.37 (95% CI=0.70–2.69); for past smokers the RR was 1.10 (95% CI=0.81–1.50), and for current smokers the RR was 1.27 (95% CI=0.79, 2.06) after additional adjustment for age at starting to smoke regularly. However, the relatively low number of lung cancer cases within each smoking category (never: 40, past: 179, and current: 104; data missing: five cases) gave limited power to detect interactions.

## DISCUSSION

In this prospective study among male health professionals, twice or more weekly aspirin use was not associated with risk of total lung cancer or NSCLC, even for long-term consistent users. Also, no dose–response association was observed for frequency of aspirin use and lung cancer incidence.

Several epidemiologic studies have reported an increased incidence of lung cancer associated with several inflammatory-related lung diseases including asthma and chronic bronchitis, suggesting that inflammation may play an important etiologic role (Wu *et al*, 1995; Mayne *et al*, 1999; Brownson and Alavanja, 2000; Brenner *et al*, 2001). The reduction of inflammatory prostaglandins by inhibition of COX-2 by NSAIDs, including aspirin, might have a chemopreventive effect. However, in a recent study of hydroxytoluene-induced early lung adenocarcinoma in mice, aspirin attenuated pulmonary inflammation but was ineffective at preventing lung tumorigenesis (Kisley *et al*, 2002). In other animal studies, COX-2 was also consistently expressed in normal bronchoalveolar and alveolar epithelium of the lung (Bauer *et al*, 2000; Wardlaw *et al*, 2000), casting some doubt on a specific role of COX-2 in the development of lung cancer.

Results from previous clinical and epidemiological studies of aspirin use and the risk of lung cancer have been mixed. An early trial among British physicians found a statistically nonsignificant 36% lower mortality rate from lung cancer among users of aspirin compared to nonusers (Peto *et al*, 1988). A prospective study based on data from the National Health and Nutrition Examination Survey found among men a statistically significant 46% lower lung cancer risk among users of aspirin during the 30-day period preceding recruitment into the cohort compared with nonusers (Schreinemachers and Everson, 1994). Two recent case–control studies (Harris *et al*, 2002; Moysich *et al*, 2002) found statistically significant reductions in lung cancer risk for aspirin users compared to nonusers. In contrast, some prospective studies reported no overall differences in lung cancer mortality (Thun *et al*, 1993) or incidence (Paganini-Hill *et al*, 1989) between aspirin users and nonusers, consistent with our results. Furthermore, in two case–control studies, lung cancer risk was not significantly lower for regular aspirin use defined as 4 days per week for 3 months compared to nonuse (Rosenberg *et al*, 1991) or seven prescriptions of aspirin and other NSAIDs received in 13–36 months before diagnosis compared to no prescriptions (Langman *et al*, 2000). In a recent case–control study, a nonsignificant reduction in total lung cancer risk (odds ratio (OR)=0.66; 95% CI=0.34–1.28, adjusted for smoking (never, past, current) and educational status (attended college, attended graduate school)) was seen among those who reported aspirin use 3 or more times per week for at least 6 months. Similar reductions in total lung cancer risk were observed for 5 or more years of aspirin use (OR=0.68; 95% CI=0.31–1.51) (Akhmedkhanov *et al*, 2002). We used a roughly similar exposure definition of aspirin use (i.e. aspirin use two or more times per week), and our findings on duration of aspirin use are in fair agreement. However, we found no significant association between aspirin use at baseline and total lung cancer incidence (RR=1.13; 95% CI=0.89–1.43, adjusted for current age, age at starting to smoke regularly, and smoking status (includes dose and time since quitting)); the risk of total lung cancer associated with consistent aspirin use for 6 years was slightly inverse (RR=0.89; 95% CI=0.47–1.67) but the results were not statistically significant.

No association was evident between regular aspirin use and NSCLC in the current study. To date, two case–control studies have examined the association between regular aspirin use and NSCLC. In one study, results for NSCLC among women taking aspirin three or more times per week for at least 6 months (adjusted OR=0.39; 95% CI=0.16–0.96) were stronger that those when all histological types combined were considered (adjusted OR=0.66; 95% CI=0.34–1.28) (Akhmedkhanov *et al*, 2002). In contrast, a second study observed similar significant risk reductions for NSCLC among men and women who were regular users of aspirin (adjusted OR=0.62; 95% CI=0.45–0.86) and total lung cancer risk (adjusted OR=0.57; 95% CI=0.41–0.78) (Moysich *et al*, 2002).

We note a number of potential limitations in the epidemiologic studies evaluating the role of aspirin and other NSAIDs in lung cancer risk, including (1) recall bias and selection bias potential in case–control studies; (2) inefficient control for smoking history; (3) small numbers of cases; and (4) misclassification due to the insufficient assessment of long-term aspirin use or lack of detailed information on aspirin dose. Although we acknowledge the dosage limitation in the current study, the lack of information on aspirin dose will result in any potential misclassification of the risk estimate towards the null. Furthermore, the current study has strengths regarding the other issues raised. The potential for recall or selection bias is greatly minimised, if not eliminated, due to the prospective study design. To minimise confounding by smoking, smoking habits were modeled to best predict lung cancer. The relative homogeneity of this population of male health professionals decreases the likelihood of residual confounding by smoking or other factor. With repeated measurements of aspirin use we account for changes in aspirin exposure over time and reduce the potential for misclassification of aspirin use. The lack of association observed between aspirin use and lung cancer risk in the present study might reflect measurement error in aspirin use. However, using our definition of regular aspirin use, we observed a markedly decreased risk of colorectal cancer and adenoma in this same population (Giovannucci *et al*, 1994), consistent with other studies. This suggests that we are able to detect important associations with aspirin use in this study. We were unable to assess the long-term use of acetaminophen and other NSAIDs due to the insufficient number of cases among users of these compounds (range of cases among users=0–8).

Our findings do not support the hypothesis that regular aspirin use is associated with a decreased risk of lung cancer. Prevention of lung cancer should be practiced primarily through smoking prevention and cessation.
